# Modeling and Analysis of Vibration Coupling in Differential Common-Based MEMS Resonators

**DOI:** 10.3390/mi16020169

**Published:** 2025-01-30

**Authors:** Jing Zhang, Zhuo Yang, Tianhao Wu, Zhichao Yao, Chen Lin, Yan Su

**Affiliations:** 1 School of Mechanical Engineering, Nanjing University of Science and Technology, Nanjing 210094, China; zhangjing@njust.edu.cn (J.Z.); yangzhuonjust@njust.edu.cn (Z.Y.); wutianhao@njust.edu.cn (T.W.); suyan@njust.edu.cn (Y.S.); 2 Navigation and Control Technology Research Institute, Norinco Group, Beijing 100089, China; njustyzc@126.com; 3 School of Engineering, Beijing Forestry University, Beijing100083, China

**Keywords:** differential common-based MEMS resonators (DCMR), vibration coupling, silicon resonant accelerometer (SRA), coupling stiffness

## Abstract

In differential MEMS resonant sensors, a pair of resonators are interconnected with other structural components while sharing a common substrate. This leads to mutual coupling of vibration energy between resonators, interfering with their frequency outputs and affecting the sensor’s static performance. This paper aims to model and analyze the vibration coupling phenomena in differential common-based MEMS resonators (DCMR). A mechanical model of the DCMR structure was established and refined through finite element simulation analysis. Theoretical calculations yielded vibration coupling curves for two typical silicon resonant accelerometer (SRA) structures containing DCMR: SRA-V1 and SRA-V2, with coupling stiffness values of 2.361 × 10^−4^ N/m and 1.370 × 10^−2^ N/m, respectively. An experimental test system was constructed to characterize the vibration coupling behavior. The results provided coupling amplitude-frequency characteristic curves and coupling stiffness values (7.073 × 10^−4^ N/m and 1.068 × 10^−2^ N/m for SRA-V1 and SRA-V2, respectively) that validated the theoretical analysis and computational model. This novel approach enables effective evaluation of coupling intensity between 5resonators and provides a theoretical foundation for optimizing device structural designs.

## 1. Introduction

MEMS (Micro-Electro-Mechanical Systems) resonators play a pivotal role as fundamental components in microsensors and oscillators across various application domains [1,2,3,4]. Differential design has been widely employed to enhance the performance of MEMS resonators [5,6,7], particularly demonstrating significant advantages in suppressing common-mode errors [8,9]. This implies that resonator devices often necessitate the deployment of pairs or multiple sets of differentially distributed resonator structures. However, in practical applications, the two resonators of differentially arranged structures, along with other connecting elements and the sensitive structures, invariably share a common substrate, such as a silicon base. These structures are briefly referred to as differential common-based MEMS resonators (DCMR). Consequently, the vibrational energy from one resonator may be coupled to another resonator through other structures or via anchor points and the substrate. This coupling phenomenon may give rise to non-negligible vibrational interference, consequently deteriorating the measurement accuracy of MEMS devices.

Prior research has long established the advantages of frequency output in MEMS resonators [10,11,12,13,14] and the effectiveness of differential design in suppressing common-mode errors [15,16,17]. The vibration coupling phenomenon, as a complex and variable factor, has been harnessed in the design of modal-localized accelerometers [18,19,20,21]. By deliberately introducing weak coupling structures between two differentially designed resonators, these accelerometers achieve substantial amplitude ratios during resonant vibration. However, for devices that rely on detecting the frequency difference output of differential MEMS resonators to reflect sensitive physical quantities, vibrational coupling may pose a significant challenge, particularly for MEMS devices that demand high precision.

The vibration coupling leads to frequency locking between the two resonators, and the resulting dead zone effect presents a major challenge in signal analysis and processing. Previous studies have demonstrated that six-state phase modulation (SSM) techniques or differential photodiode amplifiers can effectively suppress frequency coupling crosstalk [22,23]. Furthermore, applying random voltage steps to the sensor’s phase modulator can effectively suppress feedback voltage-related errors that cause dead zones, thereby mitigating dead zone effects [24]. Additionally, researchers have developed a novel error function that enables frequency analysis and compensation of sensors, successfully investigating frequency locking phenomena in sensors and providing new possibilities for improving sensor performance [25].

Against this backdrop, this paper aims to model and analyze the vibration coupling phenomenon in DCMR. Both Silicon Resonant Accelerometers (SRA) and Micromachined Silicon Vibratory Gyroscopes (MSVG) are typical sensors featuring DCMR structures. This study focuses on comparing two typical SRA structures, conducting in-depth investigations into vibration coupling interference through finite element simulation analysis and experimental verification. The paper is organized as follows: Section 1 introduces the research motivation, existing foundation, and significance. Section 2 presents the mechanical model of the DCMR system and derives the coupling stiffness calculation formula. Section 3 details the finite element modeling and simulation analysis of two accelerometer variants, SRA-V1 and SRA-V2, yielding vibration coupling curves and coupling stiffness values. Section 4 describes the experimental test system construction and analysis of vibration coupling characteristics for both structures. Section 5 presents a comparative analysis of simulation and experimental results regarding coupling intensity between resonators. Finally, Section 6 concludes the study.

This investigation can enhance understanding of MEMS resonator design and application while establishing theoretical foundations for structural optimization and vibration interference mitigation. The research outcomes contribute to achieving higher performance and stability in MEMS sensors and oscillators, specifically addressing the challenges of vibration coupling to improve measurement accuracy in MEMS devices.

## 2. Modeling of Differential Common-Based MEMS Resonator System

### 2.1. Two-Degree-of-Freedom Mechanical Model for DCMR

**Figure 1:**
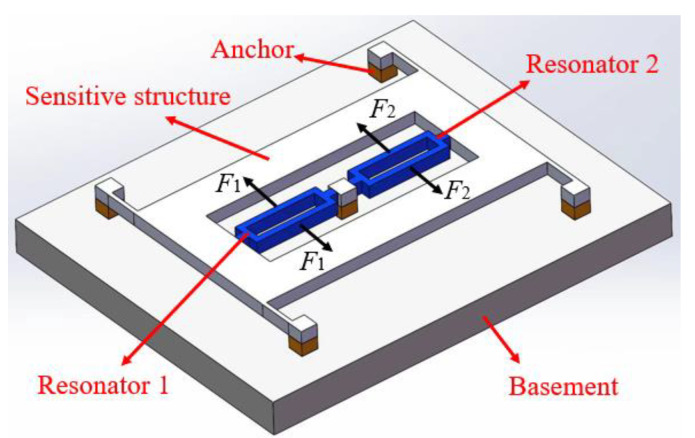
Schematic diagram of the differential common-based MEMS resonator system.

The DCMR system investigated in this work is typically encapsulated in a high-vacuum environment. As illustrated in Figure 1, the overall structure features a symmetrical design comprising a pair of differential resonators, a sensing structure (such as a proof mass for inertial force detection), multiple anchor points, and a common substrate. The pair of resonators is symmetrically connected to the substrate through the sensing structure and anchor points, indicating that both differential resonators and the sensing structure share a common base. Each resonator consists of two tuning-fork resonant beams. To mitigate common-mode errors, equal but opposite harmonic driving forces *F*_1,2_ are applied to the resonant beams, generating anti-phase forced vibrations.

**Figure 2:**
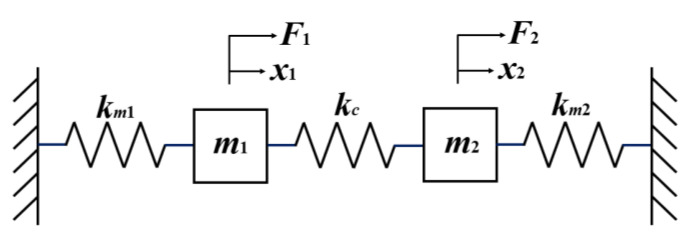
Spring-mass model of two-DOF micromechanical resonator system.

As shown in Figure 2, this DCMR system can be equivalently represented as a two-degree-of-freedom mechanical vibration model. Under high vacuum conditions, neglecting damping effects, each resonator can be simplified as a spring-mass system with spring constants *k_m_*_1_, *k_m_*_2_ and masses *m*_1_, *m*_2_. The sensing structure, anchor points, and substrate collectively form the basis for resonator coupling, which can be modeled as a coupling stiffness *k_c_*.

Applying Newton’s laws, the differential equations of forced vibration for the two-degree-of-freedom micromechanical resonator system are derived as:

(1) $$\begin{array}{l} m_{1}{\ddot{x}}_{1}=F_{1}-k_{m1}x_{1}+k_{c}\left(x_{2}-x_{1}\right)\\ m_{2}{\ddot{x}}_{2}=F_{2}-k_{m2}x_{2}-k_{c}\left(x_{2}-x_{1}\right) \end{array}$$

where *m*_1_, *m*_2_ are the equivalent masses of resonators 1 and 2, *F*_1_, *F*_2_ are the harmonic driving forces applied to resonators 1 and 2, and *x*_1_, *x*_2_ are their displacements at the driving points. Equation (1) can be written in matrix form as:

(2) $$\left[\begin{array}{cc} m_{1} & 0\\ 0 & m_{2} \end{array}\right]\left[\begin{array}{c} {\ddot{x}}_{1}\\ {\ddot{x}}_{2} \end{array}\right]+\left[\begin{array}{cc} k_{m1}+k_{c} & -k_{c}\\ -k_{c} & k_{m2}+k_{c} \end{array}\right]\left[\begin{array}{c} x_{1}\\ x_{2} \end{array}\right]=\left[\begin{array}{c} F_{1}\\ F_{2} \end{array}\right]$$

The equation of motion for the flexural vibration of the resonator beam is given as:

(3) $$x=X\sin\left(\omega t+\theta \right)$$

where *x* is the vibration displacement of the resonator beam, *X* is the vibration amplitude, *ω* = 2*πf* is the natural angular frequency of the resonator (*f* is the natural frequency), *t* is time, and *θ* is the phase angle. Substituting Equation (3) into Equation (2) yields:

(4) $$\left[\begin{array}{cc} -\omega_{1}^{2}m_{1}+k_{m1}+k_{c} & -k_{c}\\ -k_{c} & -\omega_{2}^{2}m_{2}+k_{m2}+k_{c} \end{array}\right]\left[\begin{array}{c} x_{1}\\ x_{2} \end{array}\right]=\left[\begin{array}{c} F_{1}\\ F_{2} \end{array}\right]$$

The stiffness, displacement, force matrices, and natural angular frequencies in Equation (4) are, respectively, given as:

(5) $$K=\left[\begin{array}{cc} K_{11} & K_{12}\\ K_{21} & K_{22} \end{array}\right]=\left[\begin{array}{cc} -\omega_{1}^{2}m_{1}+k_{m1}+k_{c} & -k_{c}\\ -k_{c} & -\omega_{2}^{2}m_{2}+k_{m2}+k_{c} \end{array}\right]$$

(6) $$X=\left[\begin{array}{c} x_{1}\\ x_{2} \end{array}\right],\;F=\left[\begin{array}{c} F_{1}\\ F_{2} \end{array}\right]$$

(7) $$\omega_{1,2}^{2}=\frac{2k_{c}+k_{m1}+k_{m2}}{2m}\mp \sqrt{{\left(\frac{k_{m1}-k_{m2}}{2m}\right)}^{2}+\frac{k_{c}^{2}}{m^{2}}}$$

Thus, the mathematical relationship of vibration coupling between the two resonators can be obtained. It can be seen that the coupling stiffness *k_c_* modifies the original equivalent stiffness of both resonators 1 and 2 during their vibration process to some extent. Under external driving forces ***F***, the presence of *k_c_* further affects the displacements of both DCMRs.

### 2.2. Vibration Coupling Characteristics of DCMR

To further investigate the vibration coupling characteristics of DCMR, we first examine how the coupling stiffness *k_c_* affects the amplitude-frequency characteristics of both resonators. From Equations (3)–(6), the vibration amplitudes of the two resonators can be, respectively, expressed as:

(8) $$\begin{array}{l} X_{1}=\frac{K_{22}F_{1}-K_{12}F_{2}}{K_{11}K_{22}-K_{12}K_{21}}\\ X_{2}=\frac{-K_{21}F_{1}+K_{11}F_{2}}{K_{11}K_{22}-K_{12}K_{21}} \end{array}$$

**Figure 3:**
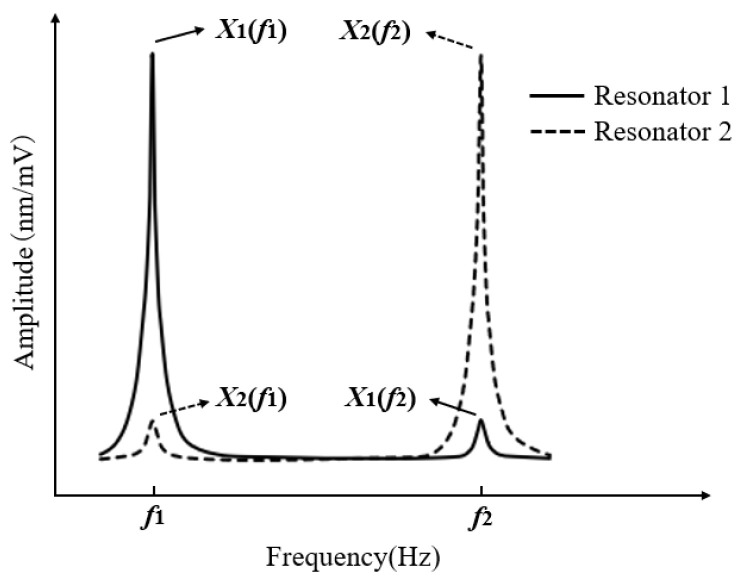
Schematic diagram of amplitude-frequency response curves for the two resonators.

Based on Equation (8), the amplitude-frequency response curves of the two resonators can be schematically illustrated as shown in Figure 3. Here, *X*_1_(*f*_1_) and *X*_1_(*f*_2_) represent the amplitude values of resonator 1 at the natural frequencies *f*_1_ and *f*_2_ of the two resonators, respectively; similarly, *X*_2_(*f*_1_) and *X*_2_(*f*_2_) represent the amplitude values of resonator 2 at the natural frequencies *f*_1_ and *f*_2_, respectively. As shown in the figure, the most significant effect of coupling stiffness *k_c_* on the resonators’ vibration displacement occurs at their respective natural frequencies *f*_1_ and *f*_2_. Even when only resonator 1 is excited, a coupled resonance peak *X*_1_(*f*_2_) can be observed at the natural frequency *f*2 of resonator 2, and vice versa.

Therefore, based on Equation (8), the amplitude ratio between the driven resonator itself and its co-based resonator at the same natural frequency can be calculated as:

(9) $$\begin{array}{l} u_{1}=\frac{X_{1}\left(f_{1}\right)}{X_{2}\left(f_{1}\right)}=\frac{\left(-4\pi^{2}f_{1}^{2}m_{2}+k_{m2}+k_{c}\right)F_{1}+k_{c}F_{2}}{\left(-4\pi^{2}f_{1}^{2}m_{1}+k_{m1}+k_{c}\right)F_{2}+k_{c}F_{1}}\\ u_{2}=\frac{X_{2}\left(f_{2}\right)}{X_{1}\left(f_{2}\right)}=\frac{\left(-4\pi^{2}f_{2}^{2}m_{1}+k_{m1}+k_{c}\right)F_{2}+k_{c}F_{1}}{\left(-4\pi^{2}f_{2}^{2}m_{2}+k_{m2}+k_{c}\right)F_{1}+k_{c}F_{2}} \end{array}$$

In Equation (9), assuming no fabrication errors, *m*_1_ = *m*_2_ = *m*. When identical harmonic driving forces are applied to both resonators, i.e.,

(10) $$F_{1}=F_{2}\neq 0$$

Combining the above conditions and taking the amplitude ratio *u*_2_ of the two resonators at frequency *f*_2_ as an example, Equation (9) can be simplified to:

(11) $$u_{2}=\frac{X_{2}\left(f_{2}\right)}{X_{1}\left(f_{2}\right)}=\frac{-4\pi^{2}f_{2}^{2}m+k_{m1}+2k_{c}}{-4\pi^{2}f_{2}^{2}m+k_{m2}+2k_{c}}$$

The amplitude ratio *u*_1_ can be analyzed similarly. Therefore, by combining Equations (7) and (11), the coupling stiffness of the DCMR system can be derived as:

(12) $$k_{c}=\frac{4\pi^{2}mu_{2}\left(f_{2}^{2}-f_{1}^{2}\right)}{1+u_{2}^{2}}=\frac{4\pi^{2}m\left(X_{2}\left(f_{2}\right)/X_{1}\left(f_{2}\right)\right)\left(f_{2}^{2}-f_{1}^{2}\right)}{1+{\left(X_{2}\left(f_{2}\right)/X_{1}\left(f_{2}\right)\right)}^{2}}$$

From Equation (12), it can be seen that the coupling stiffness *k_c_* of the DCMR can be obtained through the natural frequencies *f*_1_, *f*_2_ of the two resonators and the amplitude ratio between self-amplitude and coupled amplitude at one of the natural frequencies. In other words, the key to determining the DCMR coupling stiffness lies in obtaining the amplitude ratios of the two resonators at their natural frequencies.

## 3. Vibration Coupling Simulation Analysis of DCMR in Accelerometers

**Figure 4:**
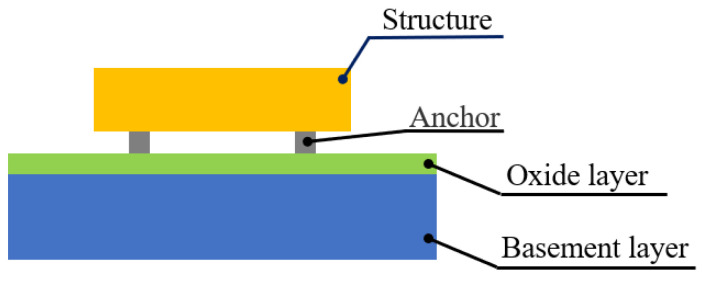
Schematic diagram of SRA structure.

The simulation analysis focuses on Silicon Resonant Accelerometers (SRA) incorporating DCMR. The cross-sectional schematic of the chip structure is shown in Figure 4, consisting of three layers: the bottom silicon substrate layer, a silicon dioxide oxidation layer in the middle, and the top layer containing anchors and the accelerometer’s sensitive structure, both made of single crystal silicon (material parameters of the structural layer are shown in Table 1). The sensitive structural layer of the SRA comprises a pair of symmetrically arranged resonators, a proof mass, a frame, and micro-levers. These components are connected to the substrate (composed of the silicon substrate layer and silicon dioxide oxidation layer) through anchors, thereby sharing a common base at all times.

**Figure 5:**
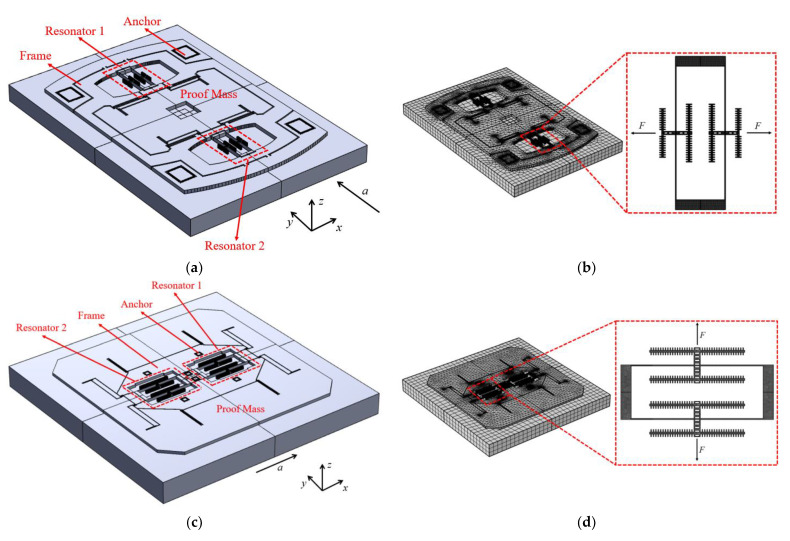
(**a**) Three-dimensional model of SRA-V1. (**b**) Mesh of SRA-V1. (**c**) Three-dimensional model of SRA-V2. (**d**) Mesh of SRA-V2.

Based on the structural characteristics described above, finite element simulation models were established for two typical SRA structures in COMSOL6.2. Figure 5a shows the three-dimensional model and mesh of the typical structure SRA-V1, while Figure 5c shows that of the typical structure SRA-V2. The thickness of each structure layer in the simulation models and the dimensions of the resonator are shown in Table 2. Both models share the same component composition in their sensitive structural layers but differ in their design layouts. In SRA-V1, the two differential resonators are distributed on both sides of the proof mass and levers, maintaining a considerable distance from each other, with all sensitive structures connected to the silicon substrate through frames and anchors on their outer sides. In contrast, SRA-V2’s differential resonators are adjacently distributed on both sides of the central anchor, with the proof mass and other sensitive structures enclosing the two resonators externally, and all anchors connecting to the silicon substrate are concentrated near the resonators. Figure 5b,d display the three-dimensional simulation models with mesh for SRA-V1 and SRA-V2, respectively, with the meshing scheme and unit size shown in Table 3.

**Figure 6:**
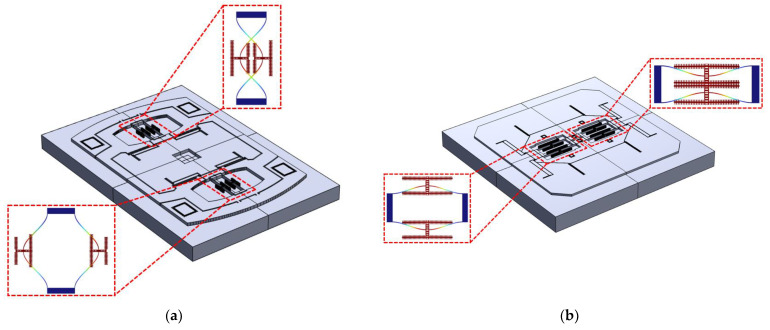
Simulation cloud diagrams of resonator vibrations under applied forces: (**a**) SRA-V1; (**b**) SRA-V2.

For the simulation of these two typical structures, in the solid mechanics analysis module, modal analysis was first performed to calculate the natural frequencies *f*_1_ and *f*_2_ of the two resonators. The resonator with the lower natural frequency was designated as No. 1 with frequency *f*_1_, while the one with the higher frequency was designated as No. 2 with frequency *f*_2_. Subsequently, frequency domain-prestressed simulation was conducted: harmonic forces *F* of equal magnitude but opposite directions were applied to the resonant beams of both resonators in the structural model. The simulation cloud diagrams of the resonator vibrations under applied forces for both structures are shown in Figure 6. Furthermore, displacement amplitude-frequency response simulation analysis was performed on the resonators, with the frequency sweep step size adjusted to ensure that the sweep frequency points included the natural frequencies *f*_1_ and *f*_2_ of both resonators. Finally, the displacement amplitude-frequency curves of both resonators in the accelerometer model were plotted.

**Figure 7:**
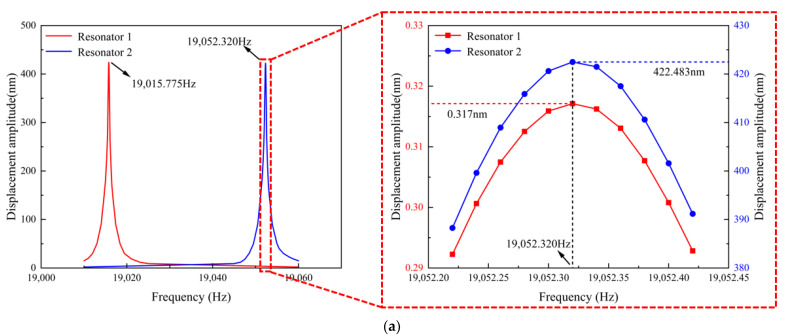

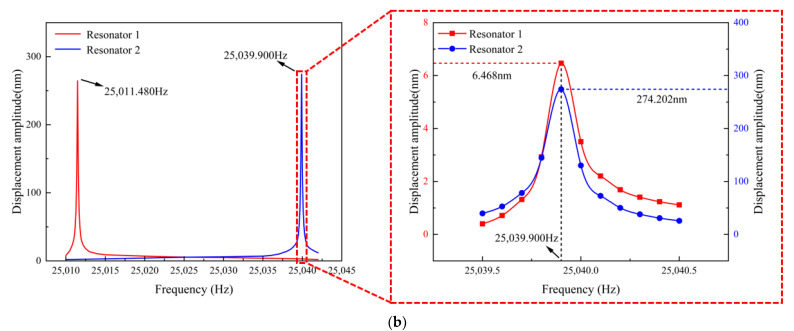
Displacement amplitude–frequency curves of the accelerometer under simulation: (**a**) SRA-V1; (**b**) SRA-V2.

As shown in Figure 7a, the displacement amplitude–frequency curves of the two resonators in SRA-V1 indicate that the natural frequencies are *f*_1_ = 19,015.775 Hz and *f*_2_ = 19,052.320 Hz. Taking the displacement amplitude ratio u_2_ of the two resonators at frequency *f*_2_ as an example, a local magnification of the displacement amplitude-frequency curves at *f*_2_ reveals that the displacement amplitude of resonator 1 at *f*_2_, *X*_1_(*f*_2_), is 0.317 nm, while the displacement amplitude of resonator 2 at its own natural frequency *f*_2_, *X*_2_(*f*_2_), is 422.483 nm. Substituting these simulation results into Equation (12) yields a coupling stiffness *k_c_* of 2.361 × 10^−4^ N/m for SRA-V1.

Similarly, as shown in Figure 6b, the displacement amplitude-frequency curves of the two resonators in SRA-V2 demonstrate natural frequencies of *f*_1_ = 25,011.480 Hz and *f*_2_ = 25,039.900 Hz. The displacement amplitude of resonator 1 at *f*_2_, *X*_1_(*f*_2_), is 6.468 nm, and the displacement amplitude of resonator 2 at its own natural frequency, *X*_2_(*f*_2_), is 274.202 nm. Using Equation (12), the coupling stiffness *k_c_* for SRA-V2 is calculated to be 1.370 × 10^−2^ N/m.

These results demonstrate that the coupling stiffness *k_c_* of SRA-V1 is only 1.723% of that of SRA-V2, indicating that the sensing structure with resonators distributed on both sides of the proof mass (SRA-V1) exhibits significantly lower coupling between resonators compared to the sensing structure with adjacent resonator distribution (SRA-V2).

## 4. Experimental Testing of Accelerometer with DCMR

**Figure 8:**
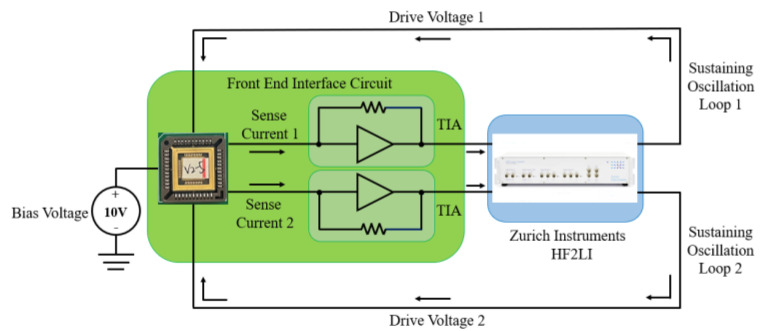
Schematic diagram of driving and detection test system for DCMR.

Figure 8 shows a schematic diagram of the test system for driving and detecting two differential resonators in the SRA. In this test system, a DC voltage source provides a 10 V bias voltage to both resonators, while an HF2LI Lock-in Amplifier supplies AC driving voltages at specific frequencies to each resonator. The weak detection current generated by each resonator is amplified by the front-end interface circuit and converted into voltage signals that are fed into the HF2LI. Using the HF2LI, the frequency and amplitude of the amplified detection voltage signals can be obtained, enabling the plotting of amplitude-frequency curves for each resonator.

**Figure 9:**
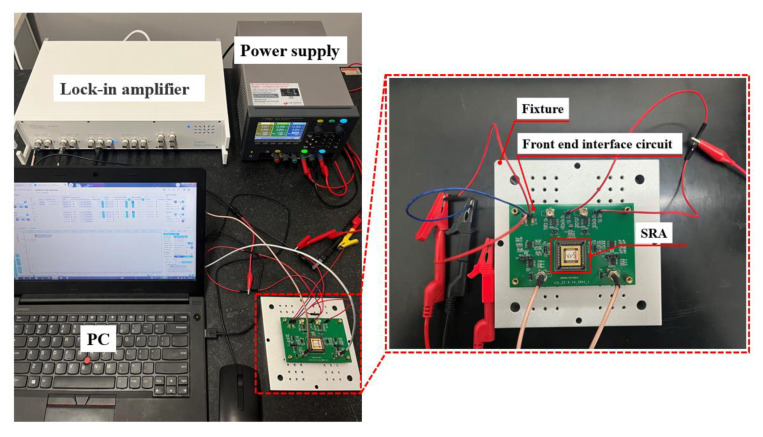
Test instruments and experimental environment.

The actual test system setup and testing environment are shown in Figure 9. The test system consists of the SRA chip, front-end interface circuit, test fixture, Lock-in Amplifier, power supply, and computer. The test fixture secures the SRA laboratory prototype on a marble platform. The Lock-in Amplifier provides AC driving voltages to the accelerometer and detects electrical signals output from the front-end interface circuit. The power supply provides power to the SRA, while the computer serves as the platform for data processing and analysis.

The resonator vibrates under the action of harmonic driving forces, and the displacement amplitude is converted into weak detection current. The conversion process is shown in Equation (13), where *I_s_* is the detection current, *K_e_* is the structural capacitance change coefficient of the resonator, and *V_bias_* is the bias voltage.

(13) $$I_{s}=K_{e}V_{bias}\dot{x}$$

The weak detection current generated by each resonator is amplified by the front-end interface circuit and converted into voltage signal (*U_s_*) by the feedback resistor (*R_TIA_*):

(14) $$U_{s}=R_{TIA}I_{s}$$

From Equations (3), (13) and (14), the conversion relationship between the maximum displacement amplitude *X* and the maximum voltage amplitude *U_s_*_max_ can be obtained:

(15) $$X=\frac{U_{s\max}}{R_{TIA}K_{e}V_{bias}\omega }$$

**Figure 10:**
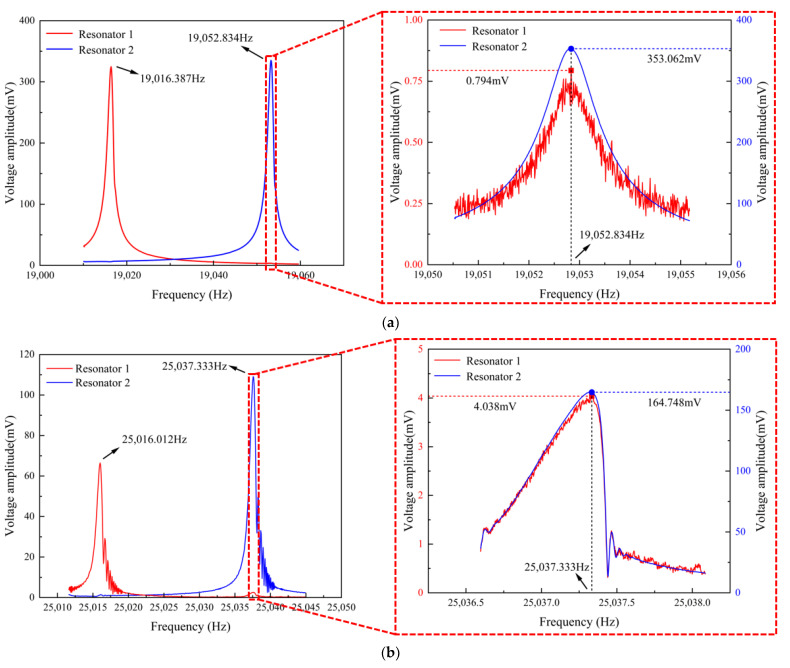
Experimental voltage amplitude-frequency curves of the accelerometer: (**a**) SRA-V1; (**b**) SRA-V2.

First, identical harmonic driving voltages were applied to both resonators of the SRA using the Lock-in Amplifier, followed by frequency sweeping through varying the driving frequency for each resonator. The voltage amplitude–frequency curves for each resonator’s detection voltage were precisely plotted by continuously adjusting the sweep range and step size. As shown in Figure 10a, from the voltage amplitude–frequency curves of DCMR in SRA-V1, since the resonator voltage amplitude reaches its maximum at its natural frequency, resonator 1’s natural frequency *f*_1_ was determined to be 19,016.387 Hz, and similarly, resonator 2’s natural frequency *f*_2_ was 19,052.834 Hz. Taking the voltage amplitude comparison at *f*_2_ as an example, a local magnification of the voltage amplitude–frequency curves at *f*_2_ shows that resonator 1’s voltage amplitude *X*_1_(*f*_2_) is 0.794 mV, while resonator 2’s voltage amplitude *X*_2_(*f*_2_) is 353.062 mV. Using Equation (15), resonator 1’s voltage amplitude *X*_1_(*f*_2_) and resonator 2’s voltage amplitude *X*_2_(*f*_2_) can be converted to displacement amplitude, which is 0.160 nm and 71.005 nm, respectively, resulting in a voltage amplitude ratio of approximately 443.781. Substituting these experimental data into Equation (12) yields a coupling stiffness kc of 7.073 × 10^−4^ N/m.

Similarly, as shown in Figure 10b, the voltage amplitude–frequency curves of the two resonators in SRA-V2 indicate natural frequencies *f*_1_ and *f*_2_ of 25,016.012 Hz and 25,037.333 Hz, respectively, with resonator 1’s voltage amplitude *X*_1_(*f*_2_) at 4.038 mV and resonator 2’s voltage amplitude *X*_2_(*f*_2_) at 164.748 mV. Using Equation (15), resonator 1’s voltage amplitude *X*_1_(*f*_2_) and resonator 2’s voltage amplitude *X*_2_(*f*_2_) can be converted to displacement amplitude, which is 0.604 nm and 24.653 nm, respectively. The voltage amplitude ratio between the two resonators is only 40.816, an order of magnitude lower than that of SRA-V1. Substituting these results into Equation (12), the coupling stiffness of SRA-V2 is calculated to be 1.068 × 10^−2^ N/m, which is 15.100 times that of SRA-V1.

Therefore, the experimentally measured coupling stiffness *k_c_* of SRA-V1 is only 6.623% of SRA-V2’s, further verifying that the coupling degree of resonators distributed on both sides of the mass block is far lower than that of adjacent resonator arrangements like in SRA-V2, consistent with the simulation conclusions.

## 5. Discussion

The simulation and experimental results for both SRA-V1 and SRA-V2 accelerometers incorporating DCMRs are summarized in Table 4. The coupling stiffness values derived from simulation and experiments are 2.361 × 10^−4^ N/m and 7.073 × 10^−4^ N/m for SRA-V1, and 1.370 × 10^−2^ N/m and 1.068 × 10^−2^ N/m for SRA-V2, respectively. While both simulation and experimental values remain within the same order of magnitude, SRA-V1 demonstrates a larger deviation between simulated and experimental results compared to SRA-V2. As illustrated in Figure 10, the vibration amplitude induced by coupling effects at the same natural frequency is relatively small and susceptible to noise interference, particularly pronounced in the measurement of resonator 1’s amplitude *X*_1_(*f*_2_) in SRA-V1. This inherent characteristic accounts for the observed discrepancies between simulation and experimental results. Nevertheless, the experimental measurements substantially validate the simulation results, confirming the effectiveness of this method in quantifying the coupling stiffness between differential resonators.

Moreover, both experimental and simulation results demonstrate that the coupling stiffness of SRA-V1 is more than one order of magnitude lower than that of SRA-V2, indicating weaker coupling between the differential resonators in SRA-V1. From a structural perspective, the fixed constraints of the two differential resonators in SRA-V1 are spatially separated, with frames and anchors positioned at the accelerometer’s periphery. In contrast, SRA-V2 features closely spaced fixed constraints, with frames and anchors located at the center of the sensing structure. Given that vibrational energy exchange between resonators occurs through fixed constraints and the substrate layer, and these constraints directly couple the sensing structure to the substrate, they serve as the primary pathway for DCMR vibration coupling. Consequently, increasing the spatial separation between anchor points and differential resonators more effectively preserves the independent vibrational characteristics of each resonator, thereby minimizing inter-resonator coupling effects.

## 6. Conclusions

This paper presents a novel method for evaluating vibration coupling phenomena in differential MEMS resonant sensors (DCMRs). Through theoretical analysis and experimental validation of two typical structures, SRA-V1 and SRA-V2, their coupling stiffness values were determined to be 7.073 × 10^−4^ N/m and 1.068 × 10^−2^ N/m, respectively, which align with the theoretical calculations (2.361 × 10^−4^ N/m and 1.370 × 10^−2^ N/m) in terms of order of magnitude. The experimental results validate the reliability of the proposed coupling stiffness calculation method, confirming its effectiveness in assessing the coupling intensity between differential resonators and its potential application in analyzing similar coupling signals.

Comparative analysis reveals that the layout of resonator fixed constraints (anchor points) is a critical factor influencing vibration coupling intensity. Positioning anchor points at the periphery of the accelerometer (as in SRA-V1) proves more effective in suppressing vibration coupling compared to central placement (as in SRA-V2), providing a crucial theoretical foundation for the structural optimization of MEMS resonant sensors. The study demonstrates that increasing the spatial separation between differential resonator fixed constraints effectively mitigates vibration coupling effects, thereby enhancing sensor performance. This finding provides clear direction for future structural optimization of MEMS resonant sensors.

## Figures and Tables

**Table 1:** The material parameters applied in the calculation.

Material	Density (kg/m^3^)	Young’s Modulus (Pa)	Poisson’s Ratio
Si	2330	1.690 × 10^11^	0.270
SiO_2_	2200	7 × 10^10^	0.170

**Table 2:** The thickness of structure layer and the dimensions of the resonator.

Dimension (μm)	Structure	SRA-V1	SRA-V2
Thickness	Structure layer	80	50
	Anchor layer	15	
	Oxide layer	2	
	Basement layer	380	
Length	Resonant beam	820	770
Width	Resonant beam of resonator 1	4.472	6.262
	Resonant beam of resonator 2	4.478	6.267

**Table 3:** Mesh information for different parts of the structural model.

Object	Meshing Scheme	Maximum Unit Size (μm)	Minimum Unit Size (μm)
Resonator	Sweep	3.700	0.500
Frame, Anchor and Proof mass	Free regular tetrahedral mesh	200	2
Oxide layer and Basement layer	Sweep	150	10

**Table 4:** The calculation results of coupling stiffness.

Accelerometer	SRA-V1		SRA-V2	
Result Source	Simulation	Experiment	Simulation	Experiment
*f*_1_ (Frequency 1)	19,015.775 Hz	19,016.387 Hz	25,011.480 Hz	25,016.012 Hz
*f*_2_ (Frequency 2)	19,052.320 Hz	19,052.834 Hz	25,039.900 Hz	25,037.333 Hz
*X*_1_(*f*_2_) (Resonator 1’s Amplitude at *f*_1_)	0.317 nm	0.160 nm	6.468 nm	0.604 nm
*X*_2_(*f*_2_) (Resonator 2’s Amplitude at *f*_2_)	422.483 nm	71.005 nm	274.202 nm	24.653 nm
*k*_c_ (Coupling Stiffness)	2.361 × 10^−4^ N/m	7.073 × 10^−4^; N/m	1.370 × 10^−2^ N/m	1.068 × 10^−2^ N/m

## Data Availability

The original contributions presented in this study are included in the article. Further inquiries can be directed to the corresponding author.
